# Significant Upregulation of Alzheimer's β‐Amyloid Levels in a Living System Induced by Extracellular Elastin Polypeptides

**DOI:** 10.1002/anie.201912399

**Published:** 2019-11-04

**Authors:** Chao Ma, Juanjuan Su, Yao Sun, Yang Feng, Nolan Shen, Bo Li, Yingxia Liang, Xintong Yang, Hui Wu, Hongjie Zhang, Andreas Herrmann, Rudolph E. Tanzi, Kai Liu, Can Zhang

**Affiliations:** ^1^ State Key Laboratory of Rare Earth Resource Utilization Changchun Institute of Applied Chemistry Chinese Academy of Sciences 130022 Changchun China; ^2^ Zernike Institute for Advanced Materials Nijenborgh 4 9747 AG Groningen The Netherlands; ^3^ Genetics and Aging Research Unit McCance Center for Brain Health MassGeneral Institute for Neurodegenerative Disease Department of Neurology Massachusetts General Hospital and Harvard Medical School Charlestown MA USA; ^4^ National Engineering Laboratory for AIDS Vaccine School of Life Sciences Jilin University Changchun 130012 China; ^5^ DWI—Leibniz Institute for Interactive Materials Forckenbeckstr. 50 52056 Aachen Germany; ^6^ Institute of Technical and Macromolecular Chemistry RWTH Aachen University Worringerweg 2 52074 Aachen Germany

**Keywords:** Alzheimer's disease, amyloid-β peptides, extracellular elastin, upregulation

## Abstract

Alzheimer's disease (AD) is a neurodegenerative disorder and the primary cause of age‐related dementia. The etiology of AD is complex and has not been completely elucidated. Herein, we report that treatment with elastin‐like polypeptides (ELPs), a component of the brain extracellular matrix (ECM), significantly increased the levels of AD‐related amyloid‐β peptides (Aβ) both in vitro and in vivo. Regarding the molecular mechanism(s), the upregulation of Aβ levels was related to increased proteolytic processing of the amyloid precursor protein. Furthermore, nesting tests demonstrated that the ELP‐treated animals showed significant neurobehavioral deficits with cognitive impairment. These results suggest that the elastin is associated with AD‐related pathological and behavioral changes. This finding presents a new aspect for Alzheimer's amyloidosis event and provides a great promise in developing ELP‐based model systems to better understand the pathogenesis of AD.

## Graphical abstract

**Treatment with elastin polypeptides**, a component of the brain extracellular matrix (ECM), increases levels of β‐amyloid, associated with Alzheimer's disease, both in vitro and in vivo. This is associated with the occurrence of early‐stage cognitive impairment in animals. This provides a new insight into Alzheimer's disease pathogenesis.

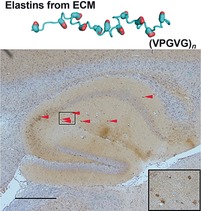

## Introduction

Alzheimer's disease (AD) is a progressive neurodegenerative disorder and the major cause of dementia in the elderly. Considerable evidence shows that upregulation and accumulation of a small peptide, called amyloid‐β (Aβ), are primary pathogenic events of AD,^1^, ^2^, ^3^ which drive the other critical components involved in AD pathologies, including tau protein hyperphosphorylation and neuroinflammation.^4^, ^5^, ^6^, ^7^, ^8^, ^9^ The etiology of AD is complex and the mechanistic elucidation of AD pathogenesis remains inconclusive. It has been challenging to comprehensively determine pathological events contributing to the onset and progression of AD.^10^, ^11^ Presently, there is an urgent need to identify and investigate new factors that may trigger Aβ overproduction in the neuronal microenvironment and to further unravel the pathogenesis of AD.

The extracellular matrix (ECM) plays an important role in regulating the development and homeostasis of cells in the central nervous system as well as other parts of human body and several therapies targeting ECM are actively being developed.^12^, ^13^ Concomitantly, the role of ECM in neuropathology has also been of growing interests. The components of ECM in the brain include basement membranes (BM) surrounding cerebral vessels and perineuronal nets (PN), as well as neural interstitial matrix (NIM).^14^ The macromolecules from the PN and NIM, such as tenascin R and proteoglycan, have been studied in the context of neurodegeneration due to their maintenance features.^14^, ^15^, ^16^ In terms of age‐related AD pathology, however, little attention has been paid to the fibrous proteins (for example, elastin) in the BM region. There are reports that elastin can be substantially fragmented and released with aging, yet the effects of these released elastin polypeptides on AD pathogenesis remain elusive.^17^, ^18^

Herein, we demonstrate that the elastin‐like polypeptides (ELPs) play a previously unrecognized role in Aβ upregulation. ELPs significantly induced the overproduction of Aβ peptides in Alzheimer's model cells (7PA2 cells, a Chinese hamster ovary cell line that stably expresses mutant human amyloid precursor protein (APP)), resulting in amounts of Aβ 5–10 times higher than those in control groups. The behavior of Aβ overproduction can be further actively tuned by temperature owing to the polymeric properties of ELPs. Furthermore, Aβ upregulation was detected in the brains of mice at two months post ELP injection. Furthermore, nesting tests demonstrated that the mice showed progressive impairment of cognitive behavior in a dose‐dependent manner. Therefore, our finding has revealed that ELPs are novel and important modulators that upregulate Alzheimer's Aβ proteins, and thus, also provide valuable knowledge to better understand the pathogenesis of AD.

## Results and Discussion

**Figure 1:**
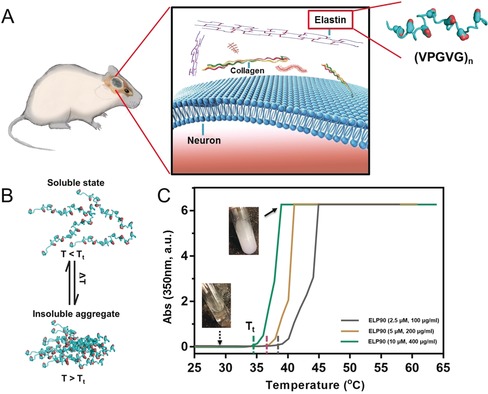
An overview of extracellular elastin. A) A schematic illustration of elastin polypeptides in the brain ECM of a mouse. Elastin is one of the major components in the ECM, consisting of repetitive sequences with low complexity. B) The typical LCST behavior of ELPs. When the temperature is higher than the transitional point (T>T_t_), soluble elastin polypeptides is triggered to form insoluble aggregates. This process is reversible. C) An investigation of absorption of ELP solution as a function of temperature. The solution stays clear until T>T_t_. The transition occurs abruptly followed by a plateau with maximum absorption, indicating the blurry phase of the solution.

The ELPs are a family of genetically encoded biopolymers and contain repeats of canonical sequences derived from native elastin. The primary structure of ELPs consists of repetitive pentapeptide units (VPGVG)n with low complexity (Figure 1 A).^19^, ^20^, ^21^ ELP30, ELP48, and ELP90 with varied lengths of polypeptide backbones were used in this study. ELP sequences and motifs dictate their physicochemical properties, for example, they are differentially responsive to external stimuli (Figure 1 B).^20^ A thermal‐related phase transition behavior, termed lower critical solution temperature (LCST), is a typical property of ELPs in aqueous environment. Soluble forms of ELPs can be triggered to become hydrophobic proteinaceous aggregates upon heating over the transitional point (T_t_). The LCST is attributed to collective factors involving abrupt changes of hydrated networks surrounding the backbone as well as reduction of water‐accessible surface area of the proteins.^20^ To quantitatively evaluate the transitional behavior, UV absorption tests at 350 nm integrated with thermal treatment were performed using ELP90 solutions (Figure 1 C). For the group with a concentration of 400 μg mL^−1^, no insoluble aggregate was observed below 34.5 °C (T_t_). When the temperature approached the critical point, a blurry phase appeared immediately, instantly reaching a plateau stage exhibiting a maximum degree of opaqueness. This phase change behavior was dependent on the concentration of ELP90. By reducing the concentration to 200 μg mL^−1^, the T_t_ shifted to 37 °C and it further increased to approximate 39 °C when the solution was 100 μg mL^−1^.

**Figure 2:**
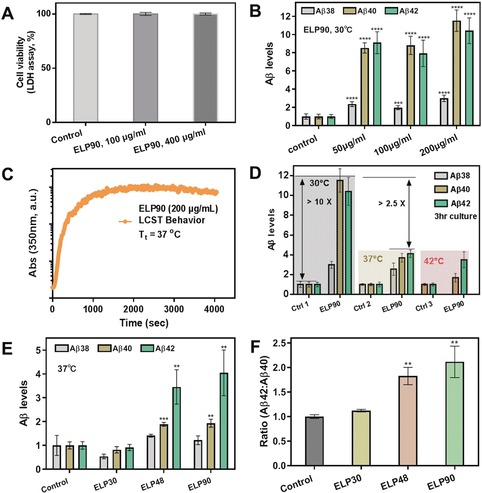
The effects of ELPs on Aβ peptides in AD 7PA2 cells (a Chinese hamster ovary cell line that stably expresses mutant human amyloid precursor protein (APP)). A) Cytotoxicity tests characterized by the LDH assay. No differences in cell viability were found when comparing ELP‐treated cells to the control, indicating excellent biocompatibility of ELPs. B) The changes of Aβ levels of 7PA2 cells cocultured with ELP90 at 30 °C. Aβ38, Aβ40, and Aβ42 showed a significant increase after treatment with ELP90 at a concentration of 50 μg mL^−1^ (p value=2.05×10^−5^, 1.66×10^−8^, and 1.59×10^−8^, vs. control, respectively), 100 μg mL^−1^ (p value=1.66×10^−4^, 4.79×10^−9^, and 4.26×10^−7^, vs. control, respectively), and 200 μg mL^−1^ (p value=3.33×10^−6^, 4.81×10^−9^, and 5.57×10^−8^, vs. control, respectively). C) The LCST profile of ELP90 at 37 °C over time. D) Overproduction of Aβs can be actively tuned by temperature when treated with ELP. At 30 °C, there was a 10‐times increase compared with controls (pure cells with PBS). The levels of Aβs were 4‐fold greater than those of the controls at 37 °C and 42 °C. E) Aβ levels can be further tuned by ELPs with varied molecular weight. Levels of Aβ40 and Aβ42 were significantly increased after treatment with ELP48 (p value=0.00084 and 0.0045, vs. control, respectively) or with ELP90 (p value=0.0020 and 0.0056, vs. control, respectively). F) The ratio of Aβ42 to Aβ40 is dependent on molecular weight of the dosed ELPs. (t‐test; mean±SD.*, *p*<0.05; **, *p*<0.01; ***, *p*<0.001; ****, *p*<0.0001.).

To investigate the interplay of ELPs and Aβ in cell lines, we utilized an AD‐model cell line termed 7PA2 cells, which has been previously reported and constitutively express familial AD mutation in the *APP* gene encoding amyloid‐β precursor protein (APP). As a first step, cytotoxicity analysis involving the lactate dehydrogenase assay (LDH) was carried out (Figure 2 A). ELP90 showed robust biocompatibility due to its proteinaceous nature and all testing groups exhibited an approximately equal ratio of cell viability (≥99 %). For the investigation of effects of ELP on Aβ levels, varied amounts of ELP90 were applied to 7PA2 cell media at 30 °C (Figure 2 B) and the Aβ levels (Aβ38, Aβ40, and Aβ42) were quantitatively evaluated by MesoScale Aβ analysis.^1^ Analysis demonstrated that Aβ oligopeptides were significantly overproduced at a concentration as low as 50 μg mL^−1^. By raising ELP concentrations first to 100 μg mL^−1^ and further to 200 μg mL^−1^, Aβ levels, particularly Aβ40 and Aβ42, dramatically increased by 10–12 fold over control groups (*p* values <0.0001). Next, the LCST behavior of ELP90 in cell culture was studied at 37 °C over time. The absorption changed abruptly in the first 300 s, followed by a long‐standing plateau at a maximum value (Figure 2 C). Under physiological conditions (*T*=37 °C) within 3 hours, levels of neurotoxic Aβ42 and Aβ40 were upregulated 4‐fold, although there was an approximately 2.5 times reduction compared with the Aβ level changes at 30 °C. We continued to investigate the interaction of ELP90 and AD cells at 42 °C and the data indicate a similar trend as the changes observed at 37 °C (Figure 2 D). Thus, the effects of ELPs on Aβ levels are associated with temperature and its aqueous environment. Thereby, this data demonstrates that the production of Aβ peptides can be significantly enhanced by ELPs in cell cultures.

Next, ELPs with varied length backbones were used to study Aβ changes in 7PA2 cells. There was an increasing trend in terms of changes in Aβ levels when the molecular weights were raised from ELP30 to ELP48 and then to ELP90 (Figure 2 E). Specifically, ELP90 showed a 4–5‐fold increase of Aβ42 in comparison to controls. Notably, the Aβ42 to Aβ40 ratio (Aβ42/Aβ40) is related to AD pathology and is a biomarker for AD.^22^, ^23^ The increasing ratio of Aβ42/Aβ40 in ELP30 groups was almost the same as controls (Figure 2 F). Alternatively, a 1.8‐fold increase was observed when comparing ELP48 to the control. This value was further enhanced to 2.2 times higher than controls when ELP90 was used (*p*<0.01). This indicates that larger molecular weights of ELPs are related to higher levels of Aβ production.

**Figure 3:**
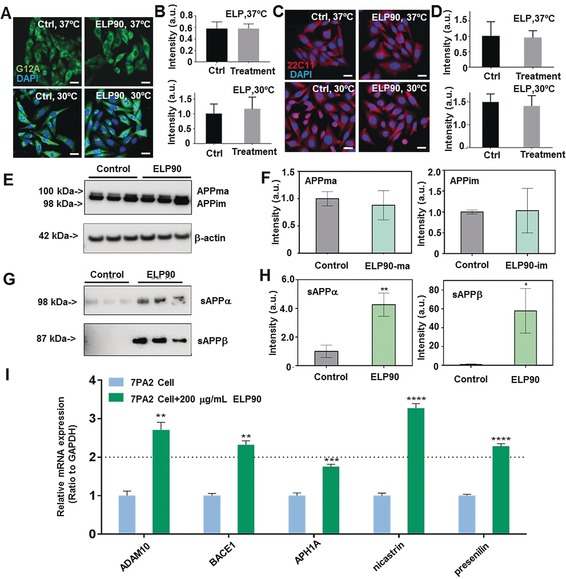
Mechanistic study of upregulation of Aβ levels. A) The representative fluorescent images for visualization of full‐length APPs in 7PA2 cells treated with PBS and ELPs at 30 °C and 37 °C. The APPs are characterized by G12A antibody and imaged in green. The cell nuclei are stained by DAPI in blue. Scale bar, 20 μm. B) The quantification of full‐length APP levels in cells shown in (A). C) The representative fluorescent images for visualization of isoforms of APP in 7PA2 cells treated with PBS and ELPs at 30 °C and 37 °C. The APP variants were imaged in red and characterized by 22C11 antibody. Scale bar, 20 μm. D) Quantitation of levels of isoforms of APP presented in (C). E) Western blot (WB) images showing full length APPs, including mature and immature types. The bands were probed with G12A. F) The quantification of APP mature and immature bands. G) The characterization of soluble sAPPα and sAPPβ with WB protocol. The control groups were the characterization of PBS‐treated cells. H) The quantification of sAPPα and sAPPβ. I) The mRNA level changes of α‐secretase subunit (ADAM10), β‐secretase (BACE1) and γ‐secretase subunits (APH1A, nicastrin, and presenilin) after treatment with 200 μg/Ml ELP90 for 24 hours were analyzed by RT‐PCR. The 7PA2 cells treated without ELP90 were controls and the mRNA levels are normalized to 1. Data from triplicate tests were collected. (t‐test; *n*=3 per each group); All data are presented as mean±S.E.M.*, *p*<0.05; **, *p*<0.01; ***, *p*<0.001; ****, *p*<0.0001.).

To investigate the underlying mechanism for this observation at a cellular level, multiple biochemical analyses were utilized, including immunofluorescence imaging, western blotting (WB), and quantitative PCR. For the immunofluorescence assay, cells were treated with antibodies G12A (targeting APP C‐terminus) and 22C11 (targeting APP N‐terminus) and characterized via confocal laser scanning microscopy (Figure 3 A–D). The two antibodies specifically recognize full‐length APP and isoforms of APP, respectively, and were coupled with secondary antibodies to show different fluorescence. The results showed that there was no significant difference between controls and ELP‐treated groups in terms of total APP (Figure 3 B,D). At different culturing temperatures (i.e., 30 °C and 37 °C), no changes in the amounts of APP were observed. Moreover, APPma and APPim, that is, mature and immature APPs, respectively, were subjected to quantitative WB analysis using the APP8717 antibody and the levels of APPma and APPim were the same as controls and no significant difference appeared (Figure 3 E,F). Collectively, this data implied that the overproduction of Aβ peptides may not result from changes in total APP profiles. The production of Aβs can be also modulated by processing of full‐length APP. Typically, APP can be digested by α‐secretases, leading to soluble forms of APPα fragments (sAPPα). β‐ and γ‐secretases sequentially cleave APP, resulting in sAPPβ and amyloidogenic Aβ peptides. Thus, we assessed the levels of sAPPα and sAPPβ from the perspective of metabolism and processing. Our results indicated that there was a significant upregulation in sAPPα levels detected by the 6E10 antibody when comparing ELP‐treated groups to the control (*p*<0.01, Figure 3 H). This also held true for the sAPPβ levels when comparing ELP‐treated groups to the control (*p*<0.05, Figure 3 H). To better understand the mechanism involving APP cleavage when dosing with ELP, mRNA levels of the secretases were quantitatively evaluated using real‐time PCR (Figure 3 I). Specific primers were designed based on the *ADAM10*, *BACE1*, *APH1A*, *nicastrin*, and *presenilin‐1* gene sequences in a *Cricetulus griseus* (Chinese hamster) genome library (see Table S3 in the Supporting Information for a full list).^24^ *ADAM10* and *BACE1* are genes encoding α‐secretase and β‐secretase, respectively. APH1A, nicastrin, and presenilin‐1 are major subunits of the γ‐secretase complex, with presenilin‐1 (PSEN1) being the catalytic subunit. GAPDH was used for normalization. The result showed that ELP90 treatment at 200 μg mL^−1^ was associated with significant upregulation of mRNA levels of the secretases mentioned above in comparison to controls. Particularly, nicastrin, one of the most important structural units of γ‐secretase, displayed a remarkable 3.5‐fold increase compared to the control (*p*<0.0001). Thus, it is possibly the overexpression of γ‐secretase that leads to substantial cleavage activities and consequently results in overproduction of neurotoxic Aβs.

**Figure 4:**
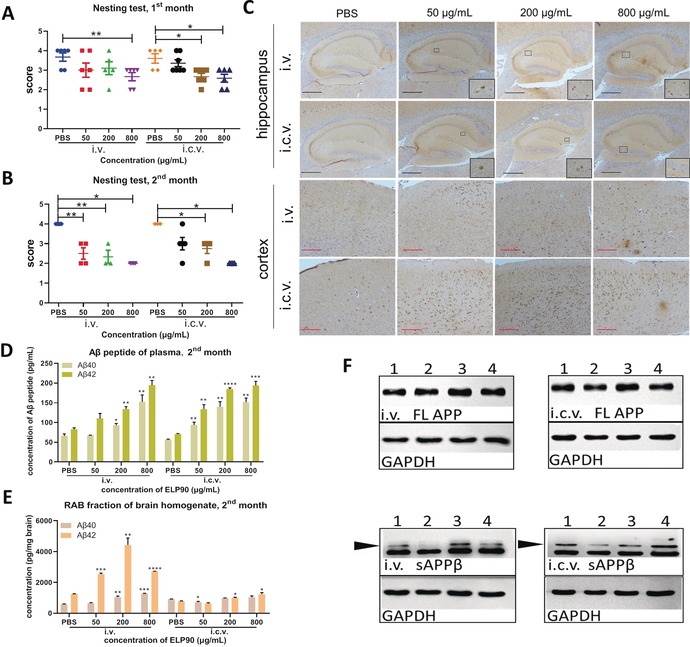
The influence of ELPs on mice. A) Results of nest‐building test (NBT) of mice 1 month after treatment. Mice in PBS subgroup show normal cognition (scored 3 to 4) in both i.v. and i.c.v. groups. The scores of ELP‐treated mice decline in a dose‐dependent fashion in the i.v. group. Particularly, the 800 μg mL^−1^ subgroup shows a significant difference compared with controls. A similar tendency occurs in the i.c.v. group, where both 200 μg mL^−1^ and 800 μg mL^−1^ subgroups show statistically significant differences to controls. n≥5 for each subgroup. Data are presented as mean±S.E.M. *p<0.05, **p<0.01, by Student's t test. B) Results of NBT 2 months after treatment. The scores of PBS subgroups indicate that the mice are in a normal cognitive state. The ELP‐treated mice showed worsened behavior. All subgroups in i.v. and i.c.v. groups (except the subgroup of 50 μg mL^−1^ in i.c.v.) showed significant differences compared to PBS‐treated mice 2 months after treatment. *n*≥3 for each subgroup. Data are presented as mean±S.E.M. *p<0.05, **p<0.01. C) Detection of Aβ amyloids in the hippocampus and cortex of mice 2 months after administration of PBS or different concentrations of ELP90. Aβ ensembles are probed by the 4G8 antibody with IHC assay in the corresponding brain regions. Compared with PBS‐treated mice, the ELP‐treated mice show a noticeable increase of 4G8 antibody immunoreactive signals in both hippocampus (marked by black frame) and frontal cortex. Scale bar: black bar 0.4 mm, red bar 0.2 mm. D) Concentration of Aβ40 and Aβ42 in plasma 2 months after treatment. The concentrations of Aβ40 and Aβ42 in all ELP subgroups were significantly higher than those in PBS subgroups (expect the 50 μg mL^−1^ i.v. subgroup). Data are presented as mean±S.E.M. *p<0.05, **p<0.01, ***p<0.001, ****p<0.0001 by Student's t test. E) Concentration of Aβ40 and Aβ42 in RAB fraction of brain homogenate 2 months after treatment. In the i.v. group, significant differences are identified in treatment groups compared with the PBS group. In the i.c.v. group, the concentrations of Aβ42 were increased in the 200 μg mL^−1^ and 800 μg mL^−1^ subgroups. Data are presented as mean±S.E.M. *p<0.05, **p<0.01, ***p<0.001, ****p<0.0001 by Student's t test. F) Western blot of sAPPβ and full length (FL) APP of RAB fraction of brain homogenate in i.v. and i.c.v groups 2 months after treatment. GAPDH acts as the internal control. Black arrows show the target bands. Lane 1: PBS group, lane 2: 50 μg mL^−1^ group, lane 3: 200 μg mL^−1^ group, lane 4: 800 μg mL^−1^ group. FL APP shows no differences among all subgroups. The levels of sAPPβ in ELP‐treated groups showed a slight upregulation compared to those of PBS control.

We further investigated the effects of ELP90 on Aβ levels and neurobehaviors using animal‐based studies. 6‐week‐old male C57BL/6 mice were utilized and treated with PBS (phosphate buffered saline) or different doses of ELP90 (50 μg mL^−1^, 200 μg mL^−1^, or 800 μg mL^−1^) via intravenous (i.v.) or intracerebroventricular (i.c.v.) injection as described in Figure S3 in the Supporting Information. Nest‐building tests have been reported to indicate cognitive changes in mice and are associated with defects in the medical prefrontal cortex and hippocampus.^25^, ^26^ Thus, we performed nest‐building tests in mice one and two months post‐ELP90 administration. The majority of mice in PBS groups were given a score of 4, indicating good cognition (Figure 4 A,B). In contrast, ELP‐treated mice showed a dose‐dependent decline in both i.v. and i.c.v. subgroups in the first month (Figure 4 A). Worsened cognitive performance was observed in the second month (Figure 4 B). We further probed protein changes in one brain hemisphere. Using immunohistochemistry staining (IHC) with 4G8 antibody, we revealed upregulated immunoreactive signals in both hippocampal and cortical areas when comparing the ELP group to the control two months post‐treatment (Figure 4 C). These evidences indicated a deteriorating tendency of cognition pathology in ELP‐treated mice over time.

Next, we performed additional biochemical analyses in plasma and the other brain hemisphere (Supporting Information, Figure S4).^27^ Enzyme‐linked immunosorbent assay (ELISA) was employed for quantitative analyses of the concentration of Aβ42 and Aβ40. In plasma, the Aβ42 levels increased after both one‐month and two‐month treatments, especially in i.v. subgroups (Figure 4 D and Supporting Information, Figure S5 F). Compared with the PBS group, the concentrations of Aβ42 in the RAB fraction (aqueous fraction) of the brain homogenate were significantly increased in the first month, especially in the groups treated with i.v. approach (Supporting Information, Figure S5 C). These differences were further enlarged after two‐months of treatment (Figure 4 E). A similar trend was observed in the RIPA fraction (detergent‐soluble fraction, Supporting Information, Figure S5 A,D), but no statistical differences were detected in the FA fraction comparing ELP‐treated groups to the control (insoluble fraction, Figure S5B and S5E). Furthermore, we investigated the levels of sAPPβ by WB. The levels of sAPPβ appeared to be elevated in the RAB fractions of brain homogenates, comparing ELP‐treated groups to PBS‐treated groups. We found that the levels of sAPPβ in RAB fractions from 50 μg mL^−1^‐dosage animals in both i.v. and i.c.v. groups did not differ compared to the PBS treatment groups after two‐months of treatment. In contrast, the levels in the RIPA fractions showed an approximately 10 % increase (Figure 4 F and Supporting Information, Figure S6). Thereby in total, the amounts of released sAPPβ were increased. Notably, the levels of full length (FL) APP showed no differences in either RAB or RIPA fractions during the whole observation period (Figure 4 F and Supporting Information, Figure S6). Thus, this animal‐based evidence supported those from cell‐based investigations, suggesting that ELPs administration results in increasing Aβ levels.

Upregulation of Aβ levels may drive other downstream AD‐related pathologic events, including tau hyperphosphorylation. We have shown that administration of ELP90 induced a robust increase of Aβ levels in both cell and animal models. Next, we investigated whether ELP may affect tau protein hyperphosphorylation using IHC and focusing on the hippocampal CA3 region. We showed that the pTau (phosphorylated at Ser396) positive immunostaining signals were increased in both the soma and dendrite of CA3 hippocampal neurons in animals in the second month after ELP treatment compared to the control animals.^28^ This indicated an increase in tau phosphorylation when the mice were treated with ELPs (Supporting Information, Figure S7). Together, these results show that there are multiple lines of molecular pathologic processes in ELP‐treated animals, including upregulation of Aβ levels as well as increases in tau phosphorylation, which collectively affect AD‐related pathological and neurobehavioral progression in these animals.

## Conclusion

Here, we investigated the interplay of extracellular elastin proteins and Alzheimer's Aβ peptides both in vitro and in vivo. It is demonstrated that ELPs can significantly upregulate Aβ proteins. Particularly, exposure to ELPs can lead to robust increases in Aβ levels up to ten times higher than controls using mechanisms that involve upregulation of APP proteolytic processing. Furthermore, treatment with ELPs in animals results in both AD‐related pathological and neurobehavioral changes, including upregulation of Aβ levels and cognitive impairment. Collectively, our results suggest that the elastin from brain ECM may be a key player in initiating the neurobehavioral defects and pathological changes underlying AD. Our data also strongly suggest that ELPs, and potentially other relevant biomaterials, should be further investigated, which may serve as valuable model systems to better modulate and completely elucidate the pathogenesis of AD.

## Conflict of interest

The authors declare no conflict of interest.

## Supporting information

As a service to our authors and readers, this journal provides supporting information supplied by the authors. Such materials are peer reviewed and may be re‐organized for online delivery, but are not copy‐edited or typeset. Technical support issues arising from supporting information (other than missing files) should be addressed to the authors.

Supplementary
