# Supplementary material for: Significant Upregulation of Alzheimer's β‐Amyloid Levels in a Living System Induced by Extracellular Elastin Polypeptides
Source: Angew Chem Int Ed Engl. 2019 Nov 4;58(51):18703–9. doi: 10.1002/anie.201912399 (PMC7187254; doi:10.1002/anie.201912399)
Supplement: Supplementary file 1 — Supplementary [file ANIE-58-18703-s001.pdf]

Angewandte Chemie
Eine Zeitschrift der Gesellschaft Deutscher Chemiker

Supporting Information

Significant Upregulation of Alzheimer's β-Amyloid Levels in a Living System Induced by Extracellular Elastin Polypeptides

Chao Ma[+], Juanjuan Su[+], Yao Sun[+], Yang Feng, Nolan Shen, Bo Li, Yingxia Liang, Xintong Yang, Hui Wu, Hongjie Zhang, Andreas Herrmann, Rudolph E. Tanzi, Kai Liu,* and Can Zhang**

anie_201912399_sm_miscellaneous_information.pdf

<u>Supplementary Material</u>

Significant upregulation of Alzheimer's β-amyloid levels in living system induced by extracellular elastin polypeptides

1. Materials

Major chemicals, kits and antibodies used in this study are listed in table S1 and table S2. Water (typically 18.2 MΩ·cm at 25 °C) was from a Milli-Q ultrapure water system (Merck, Germany). Reagents for ELP expression, such as LB medium, salts, antibiotics as well as inducer compounds, were used as received (from Sigma-Aldrich) without any further purification. *E. coli* XL1-Blue competent cells for plasmid amplification were purchased from Stratagene (La Jolla, CA). Oligonucleotides for sequencing were from Sigma-Aldrich (St. Louis, MO). Alpha-cyano-4-hydroxycinnamic acid was used as matrix during MALDI mass spectrometry and was purchased from Thermo Scientific (Waltham, MA). Animal experiments were in agreement with the guidelines of the Regional Ethics Committee for Animal and Clinical Experiments of Jilin University Institutional Animal Care and Use and the Second Hospital of Jilin University, respectively. Other reagents used in the work are analytical grade.

2. ELP Preparation

Protein expression and purification

The expression vectors for ELP30, ELP48 and ELP90 were from addgene.org (#67014, #68395, #68392). *E. coli* BLR (DE3) cells (Novagen) were transformed with expression vectors containing the ELP genes. For protein production, Terrific Broth medium (for 1 L, 12 g tryptone and 24 g yeast extract) enriched with phosphate buffer (for 1 L, 2.31 g potassium phosphate monobasic and 12.54 g potassium phosphate dibasic) and glycerol (4 mL per 1 L TB) and supplemented with 100 $\mu g{\cdot}mL^{-1}$ ampicillin, was inoculated with an overnight starter culture to an initial optical density at 600 nm (OD600) of 0.1 and incubated at 37°C with orbital agitation at 250 rpm until OD600 reached 0.7. Protein production was induced by a temperature shift to 30 °C. Growth of cultures was continued for additional 16 h post-induction. Cells were subsequently harvested by centrifugation (7,000 × g, 20 min, 4 °C), resuspended in lysis buffer (50 mM sodium phosphate buffer, pH 8.0, 300 mM NaCl, 20 mM imidazole) to an OD600 of 100 and disrupted with a constant cell disrupter (Constant Systems Ltd., Northands, UK). Cell debris was removed by centrifugation (40,000 × g, 90 min, 4 °C). Proteins were purified from the supernatant

under native conditions by Ni-sepharose chromatography. Product-containing fractions were pooled and dialyzed against ultrapure water and purified by ion-exchange chromatography using Q-HP as well as EndoTrap-HD columns. Protein-containing fractions were dialyzed extensively against ultrapure water. Purified proteins were frozen in liquid N_2, lyophilized, and stored at -20 °C until further use.

Characterization of ELPs

The concentrations of purified polypeptides were able to be determined by measuring absorbance at 280 nm using a spectrophotometer due to the existence of a Trp/Tyr residue at the C-terminus of the protein (Spectra Max M2, Molecular Devices, Sunnyvale, USA). Product purity was determined by sodium dodecyl sulfate polyacrylamide gel electrophoresis (SDS-PAGE) on a 10% polyacrylamide gel. Afterwards, gels were stained with Coomassie staining solution (40% methanol, 10% glacial acetic acid, 1 $g{\cdot}L^{-1}$ Brilliant Blue R250). Photographs of the gels after staining were taken with an LAS-3000 Image Reader (Fuji Photo Film GmbH, Düsseldorf, Germany). The staining result is shown in Figure S2.

Protein characterization employing mass spectrometry

Mass spectrometric analysis was performed using a 4800 MALDI-TOF Analyzer in linear positive mode. The protein samples were mixed 1:1 v/v with Sinapinic Acid matrix (SIGMA) (100 $mg{\cdot}mL^{-1}$ in 70% MeCN and 0.1% TFA). Mass spectra were analyzed with the Data Explorer software (version 4.9).

3. Studies at Cellular Levels

Cell Culture and Cell Lysis

The 7PA2 cells Chinese hamster ovary (CHO) cell line stably overexpress APP carrying the Indiana mutation. The expressed wild type protein was APP751 and the cells were transiently transfected with the GFP-PS1-RFP FRET reporter.[1,2] Cells were cultured in Dulbecco's modified Eagle's medium (DMEM) containing 10% fetal

bovine serum (FBS), 200 μg ml^{-1} G418, 2 mM L-glutamine, 100 μg mL^{-1} streptomycin, and 100 μg mL^{-1} penicillin. 7PA2 cells were lysed in M-PER++ solution which consisted of Mammalian Protein Extraction Reagent and EDTA/Trypsin. The lysates were collected and centrifuged at $13x10^3$ g for 20 min in the 4 °C and the supernatants were transferred into new Eppendorf tubes.

MesoScale Aβ analysis

4G8 MesoScale Aβ 3-plex kits were used to measure Aβ38, Aβ40, and Aβ42 levels in 96-well-based assay plates. Briefly, the plates were blocked with diluent and incubated with shaking for 1 h. 25 μL of detection antibody was added to the 96-well plates. Next, 25 μL MesoScale protein standards and the samples were added into the above plates and the plates were incubated on the shaker for 2 h. Next, the electrochemi-luminescence signals of the standard and protein samples were read by the MesoScale SQ 120 system (Meso Scale Diagnostics LLC, USA). Finally, the Aβ38, Aβ40, and Aβ42 levels were analyzed by the MesoScale protein standards.

Homogeneous Membrane Integrity Assay

50 μL of cell culture supernatant was added to 96 well plates, and 50 μL of CytoTox-ONE™ Reagent was added into the cell culture supernatant. Next, the solution was mixed at room temperature and incubated in the 37 °C temperate box for 30 min. The fluorescence signals were recorded with the excitation wavelength of 560 nm and the emission wavelength of 590 nm.

Immunocytochemistry

75,000 7PA2 cells per well were seeded into the chamber slides overnight. 100 μg mL^{-1} ELP solution was added into the chamber slides for 3 h. Next, the slides were washed using PBS 3 times for 5 min each. Then, the 7PA2 cells were covered with formaldehyde diluted (4%, 200 μL per well) at room temperature for 20 min on the rocker. Slides were rinsed 3 times using PBS for 5 min each and blocked in blocking buffer for 1 h. Next, 200 ul of primary antibody was added per well of slides, and

incubated for 2 h at room temperature. Slides were rinse 3 times using wash buffer for 5 min each and incubated in blocking buffer and secondary antibody at room temperature for 45 min on the rocker. Cells were washed with PBS 5 times for 10 secs each and then incubated with 20 μg/mL of DAPI solution for 20 min, and washed with PBS 5 times for 10 min each. Finally, the chambers were removed, the slides were dried at room temperature for 30 min, and stored in the dark at 4 °C until imaging.

4. Studies at Animal Levels

To investigate whether ELP could induce AD-like pathology and symptoms in mice, 57 male mice (C57BL/6, 8-week-old) were employed for in vivo experiments. Four groups were administrated, via intravenous injection (i.v.), with three doses of ELP90 at one dose per day over three consecutive days (100 μL/dose): PBS (n=6), 50 (n=6), 200 (n=5), 800 (n=5), and four groups were administrated via intracerebroventricular injection (i.c.v.) with one dose of ELP90 (1 μL/dose): PBS (n=5), 50 (n=7), 200 (n=6), 800 (n=6). In the i.c.v. group, all mice were anesthetized with 10%(w/V) sodium pentobarbital, administered in the hippocampal area with a stereotactic positioning system. A small hole was drilled a in the right hemisphere of the skull, then a micro-syringe (catalog 80383, Hamilton, Switzerland, needle size 30s) was slowly inserted the into brain at a coordinate of x:1.5mm, y:-1.7mm, z:1.8mm from bregma, and 1μL PBS or ELP90 was injected into the CA1 region of the hippocampus. Following the injection, the needle was slowly pulled out and the wound was sutured. Mice were placed in a warm environment until palinesthesia.

Nest building tests were begun at the 30th day and the 60th day after administration of ELP90 or PBS. After grading the nest building test, blood samples of all mice were taken from the submandibular vein. Blood was collected into 1.5mL EDTA EP tubes, centrifugated under 1200g in 4 °C for 20min, and the supernatant were divided as plasma samples and transferred in -80 °C.

Two mice of each group were sacrificed following the nest building test on the 30th day, and the remaining mice were sacrificed after the second nest building test. The mice were deeply anesthetized by 10% (w/V) of sodium pentobarbital, then perfused with 15mL 0.9% NaCl. Brains were taken out and divided into two hemispheres at the sagittal suture, the right hemispheres were immersed in 4% paraformaldehyde for the following IHC test, and the left hemispheres were quick-frozen with liquid nitrogen and transferred to -80 °C for brain homogenate examinations.

All animals were housed in Individually Ventilated Cages (IVC) in animal experimental platforms, core facilities for life science, Jilin University, which were maintained three per cage and at 20-22 °C on a 12-hour dark/light cycle in 40-60% humidity. All experiments were performed in accordance with legal and institutional guidelines and were approved by the Ethical Committee of Care and Use of Laboratory Animals at Jilin University.

Nest Building Test

The Nest Building Test is one of the methods used to evaluate the cognitive state of mice. The tests began in the evening (17:00-18:00), all the mice was allocated to a single cage, together with new aseptic sawdust and two pieces of 5-centimeter square cotton in the center of the cage, and then the lights were turned off. After 16 hours three trained experimenters, who were given no information about the groups, graded all the mice independently . A score of 1 indicates that the cotton was not noticeably touched, a score of 2 indicates that the cotton was partially torn up, a score of 3 indicates that the nest was partly finished and lower than the mouse’s head, a score of 4 indicates that the nest was perfect or nearly perfect. The average scores of individual mice were calculated. The p-value were analyzed by Student T-test. Mann-Whitney U rank sum test were applied in non-normal distribution data.

Immunohistochemistry (IHC)

One brain hemisphere of each mouse was fixed with 4% paraformaldehyde for two

days, then gradient dehydrated with alcohol from a concentration of 70% to 100%. Samples were transparentized with dimethylbenzene twice for a total time of 5 hours, followed by wax immersion and paraffin embedding. Ten consecutive 5μm thick sagittal sections cut approximately 600-700 μm from the sagittal suture were used to examine Aβ plaques, phosphorylated tau and microglia. Succinctly, paraffin sections were submerged in citrate buffer (pH 6.0) and boiled for 3min in a pressure cooker to expose antigen, then blocked by hydrogen peroxide and 10% goat serum sequentially. Diluted primary antibody was placed onto brain sections and incubated at 2-8 °C overnight. Slides were washed with TBST (Tris 20 mM, NaCl 137mM, 0.2% Tween-20, pH=7.2) then Primary Antibody Amplifier (UltraVision Quanto Detection System HRP DAB, thermo, CA) was applied and incubated for 30 min at room temperature, followed by HRP Polymer for another 30 min. Coloration was with DAB Chromogen for 1 min and hematoxylin staining after washing with water, then sealed with neutral gum. Brain sections were imaged by Nikon eclipse T1-S, and photo'd by Nis Elements D 4.00.00.

<u>Preparation of Brain Homogenate</u>

One brain hemisphere of each mouse was deep frozen in liquid nitrogen just after being taken out, and then transferred to -80℃ before homogenizing for western blot and ELISA detection. Brain samples were weighed and homogenized in 15μL RAB buffer per milligram (ReAssembly Buffer, 100mM MES, 1 mM EDTA, 2 mM dithiothreitol, 0.75 M NaCl, 0.5Mm MgSO4, pH 6.8) with protease and phosphatase inhibitor MIX (1mM PMSF, 10mM sodium fluoride, 1mM sodium pyrophosphate, 1mM sodium orthovanadate) in a Bead Mill Homogenizer (Bead Ruptor 24 Elite, OMNI International, Inc., 935-C Cobb Place Boulevard Kennesaw, GA 30144) at a speed of 5.65m/s for 45s with a 30s pause between two cycles. Then 1mL brain homogenate suspension was transferred to a 1.5mL microfuge tube (357448, Beckman Coulter, Inc., 250 S Kreamer Bivd Brea, CA 92821) and centrifuged at 50000g for 20 min at 4℃ (Optima Max-XP, Beckman Coulter, Inc., CA). The supernatant was saved as RAB-soluble fraction (aqueous fraction). The pellet with

resuspended in RAB buffer and centrifuged, and the supernatant was discarded. The pellet was resuspended with 1mL RIPA buffer (Radio ImmunoPrecipitation Assay, 150mM NaCl, 50mM Tris, 25mM EDTA, 1% Triton X-100, 0.5% deoxycholic and 0.5%(w/v) SDS, (pH 8.0)) with protease and phosphatase inhibitor MIX and centrifuged at 50000g for 20 min at 4℃ to collect the supernatant as the RIPA-soluble fraction (detergent soluble fraction). The pellet was suspended again with RIPA buffer, centrifuged and the supernatant was discarded. Then the pellets were further dissolved by 0.5mL 70% formic acid in 2-8℃ overnight as FA-soluble fraction (insoluble fraction). The three fraction samples were stored in Eppendorf tubes followed by cryopreserved in -80℃ before detection to avoid freeze/thaw.

Enzyme-linked immunosorbent assay (ELISA)

To monitor Aβ40 and Aβ42 in the plasma and brain homogenate of mice, ELISA kits for mouse Aβ40 (catalog CEA864Mu) and Aβ42 (catalog CEA946Mu) were employed. Plasma or brain homogenate samples of a single group were mixed in a ratio of 1:1 as a mixture sample for detection. Duplicate plasma samples were diluted and brain homogenate was 50-fold diluted before experiments. The concentration of Aβ40 and Aβ42 of plasma or brain samples were detected as described in the kit manual. Triplicate samples were used and the concentration was calculated according to the standard curve.

Western Blotting Analysis

Western blotting analysis has been described in previous work.[3] Briefly, equal amounts of fresh protein or brain homogenate were used for electrophoresis by the Novex NuPAGE SDS-PAGE Gel System. Then, the protein was transferred to membranes, and the membranes were blocked and incubated with antibody (G12A, sAPPβ, 6E10, and β-actin). β-actin or GAPDH antibody were used as an internal control. Finally, the membranes were developed using LI-COR Odyssey Fc imaging system (LI-COR, Lincoln, NE, USA). The analysis of protein used ImageJ (NIH, Bethesda, MD, USA) and Image Studio (LI-COR, Lincoln, NE, USA) software.

Real-time reverse transcription polymerase chain reaction (RT-qPCR)

RT-qPCR was used for quantification of mRNA of 7AP2 cell after treatment with ELP90, and indicated the expression level of α- secretase (ADAM10 domain), β-secretase (BACE1), γ-secretase complex (APH1A subunit, nicastrin subunit and presenilin-1 subunit) and glyceraldehyde-3-phosphate dehydrogenase (GAPDH). Briefly, 5×10^5 cell/well were added in 6 well plate, plates were incubated at 37◦C in an incubator with 5% CO_2 for 24h, then the culture media was aspirated and 2mL of fresh DMEM was added so that there was a final concentration of 200μg/mL ELP90 and incubation continued for another 24 hours. Cells that were not treated with ELP were used as a control. Cells were digested with 0.25% trypsin for 2 min at 37 °C, then FBS was added to inactive trypsin. Collected cells were washed twice with PBS. Total RNA was extracted from 7AP2 cells (control) and the 7AP2cell + ELP90 cells with Trizol® Reagent (Invitrogen) according to the manufacturer's specifications. The quality and quantity of the RNA were verified by optical density reading using a NanoDrop 200c spectrophotometer (Thermo Scientific). 1 μg of total RNA was used for cDNA synthesis in a total of 20μL with TransScript® All-in-One First-Strand cDNA Synthesis SuperMix for qPCR (Trans), including 4μL TransScript® All-in-One SuperMix for qPCR, 1μL gDNA Remover, and variable RNase-Free Water. Real-time PCR was determined with a CFX96 Real-time System (Bio-Rad) with a 20μL PCR reaction mixture that included 10μL 2×SG Fast qPCR Master Mix (BBI Life Sciences), 0.4μL of 10μM forward primer, 0.4 μL of 10 μM reverse primer, 2μL DNF buffer, 0.5 μL of cDNA and 6.7 μL PCR-grade water. Reaction conditions were 95 °C for 3 min, then 40 cycles of 95 °C for 3s and 60 °C for 30s. Triplicate samples were used for each target protein. At the end of the PCR cycles, melting curve analysis was used to monitor specificity of the PCR products. The negative control reactions contained nuclease-free water instead of template. The length of the products are designed to be between 100bp and 300bp. Relative expression levels of the selected target genes were calculated with the CFX Manager software method. The cycle threshold (Ct) values determined for the target genes were normalized against the reference gene. All PCR fragments were analyzed on a DNA1000 Labchip

(Agilent Technologies) to check amplification of a single product and sequenced to verify the obtained PCR product. Primers for each selected gene were designed using primer-BLAST software (www.ncbi.nlm.nih.gov/tools/primer-blast/) and listed in Table S3.

Table S1. The chemicals used in this study.

chemicals and kits	source	identifier
ELISA kit for mouse Aβ40	Cloud Clone Corp.	CEA864Mu
ELISA kit for mouse Aβ42	Cloud Clone Corp.	CEA946Mu
UltraVision Quanto Detection System HRP DAB（IHC kit）	thermo	TL-060-QHD
Trizol® Reagent	Invitrogen	15596018
TransScript All-in-One First-Strand cDNA Synthesis SuperMix for qPCR(One-Step gDNA Removal)	transgene biotech	AT341-01
2X SG Fast qPCR Master Mix (High Rox)	sangon biotech	B639273
DAPI	Sigma-Aldrich	D9542
CytoTox-ONE™ Homogeneous Membrane Integrity Assay	Promega corporation	G7891
V-PLEX Aβ Peptide Panel 1 (4G8) Kit	MESO SCALE DISCOVERY	K15199E-4
Fetal Bovine Serum	Gibco	10082147
Dulbecco's Modified Eagle Medium (DMEM)	Lonza BioWhittaker	BE12-614F
Trypsin/EDTA (1X) Solution	Lonza BioWhittaker	BE17-161E
Corning® 100 mL G418 Sulfate, Liquid, 50 mg/ mL Solution	Corning	30-234-CI
Corning® 100 mL L-Glutamine	Corning	25-005-CI
Corning® 100 mL Penicillin-Streptomycin Solution, 100x	Corning	30-002-CI

Table S2. Antibodies used in cell and animal experiments.

antibodies	source	identifier	ratio
4G8，anti-β-amyloid 17-24	Biolegend	800701	1:500
Anti-Phospho-Tau (Ser396) mouse monoclonalantibody (PHF13)	Biolegend	829001	1:500
Anti-GAPDH mouse monoclonal antibody	Proteintech	60004-1-lg	1:10000
Anti-Alzheimer precursor protein(APP) A4 mouse monoclonal antibody (22C11)	Merck Milipore	MAB348	1:1000
Anti-Human Synthetic peptide in portion of C-terminus of Human sAPPβ-Wild Type (target sequence: ISEVKM)	IBL	18957	2 μg/mL
Peroxidase-conjugated AffiniPure goat anti-rabbit IgG(H+L) antibody	Jackson ImmunoResearch	111-035-144	1:100000
Peroxidase-conjugated AffiniPure goat anti-mouse IgG(H+L) antibody	Jackson ImmunoResearch	115-035-003	1:100000
anti beta actin antibody	Cell Signaling	4967s	1:1000
Anti-Aβ1-16 mouse monoclonal antibody (6E10)	Biolegend	803015	1:1000
G12A antibody (targeting APP-C terminus）	Custom-manufactured by Thermo Fisher Scientific Inc		1:1000 for Western Blot; 1:400 for ICC

Table S3. All primers designed for RT-qPCR experiments.

primer	sequence
GAPDH-F	GCTCTCTGCTCCTCCCTGTTC
GAPDH-R	CAATACGGCCAAATCCGTTCAC
ADAM10-F	CGCGGTTAACCCGTGAGGA
ADAM10-R	TTTCCATACTGACCTCCCAGC
BACE1-F	GAGACCTCCGAAAGGGTGTG
BACE1-R	ACCAGCGAGTCAAAGAAGGG
nicastrin -F	GCTGGATAGCCGGTCCTTTT
nicastrin -R	ATGTTCTCTAGCCGCACAGG
presenilin-1-F	CCTTCGAGGTCCTTAGGCAG
presenilin-1-R	GTCAAGTCTCCGCCTGTCAT
Aph1a-F	TGTCCGTGACCATTAGCACC
Aph1a-R	TAGAAAACCTCCCCAGCCCT

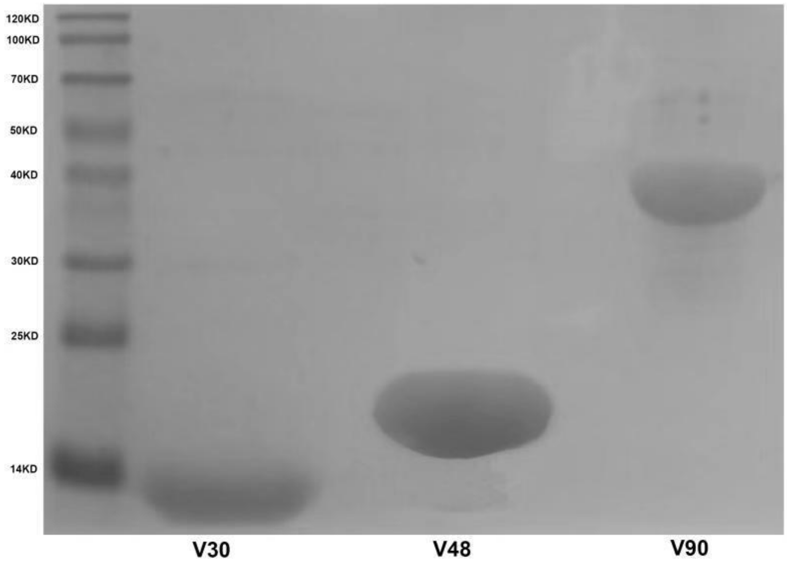

Figure S1. ELP samples (ELP30, ELP48 and ELP90) in this study were characterized by SDS-PAGE.

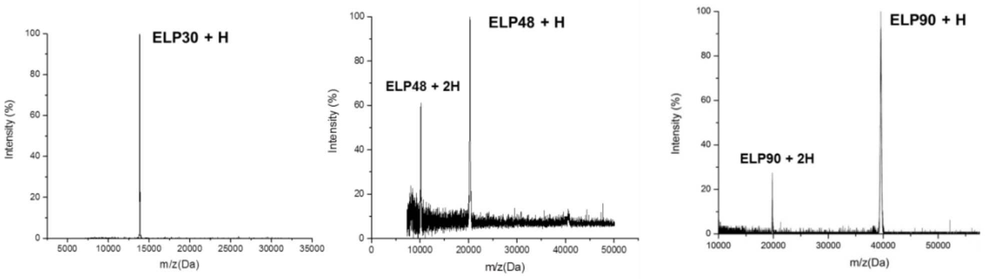

Figure S2. MALDI-TOF mass spectra of the ELP samples.

Mass determination of supercharged proteins. *average molar mass calculated with ProtParam tool. #molar mass determined by MALDI-TOF mass spectrometry.

	$M_{calculated}$* (Da)	M_{MS}# (Da)
**ELP30**	13828	13829 +/- 50
**ELP48**	20024	20086 +/- 50
**ELP90**	39671	39676 +/- 50

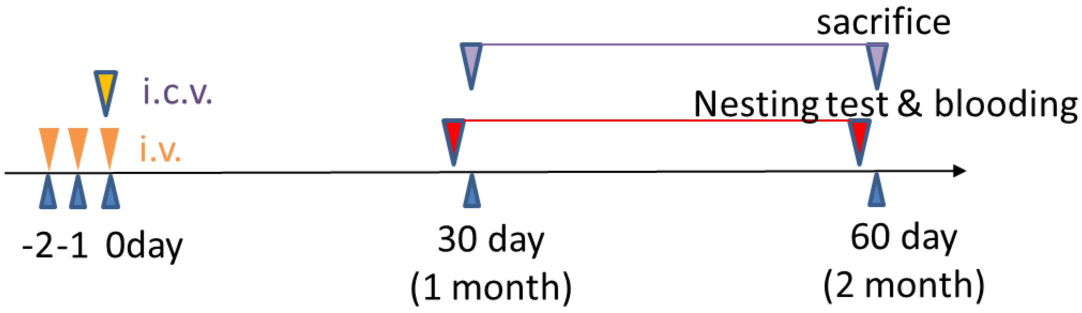

Figure S3. The timeline of the animal tests.

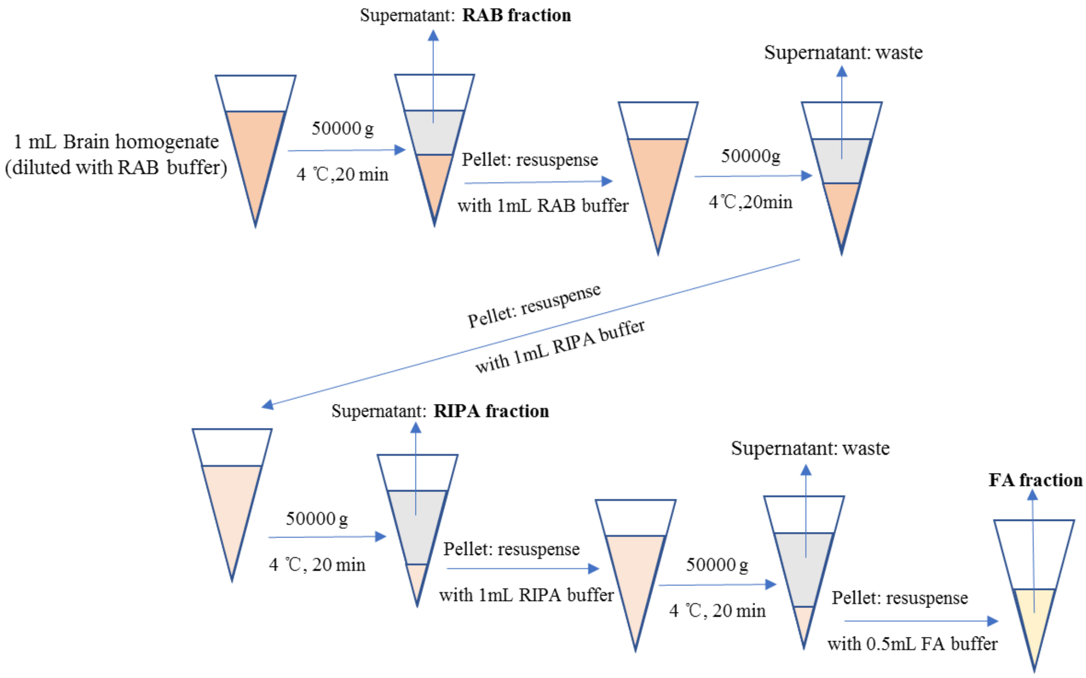

Figure S4. A scheme of the processing of mouse brain homogenates. Brain proteins were extracted progressively by RAB buffer (aqueous fraction), RIPA buffer (detergent soluble fraction) and FA buffer (insoluble fraction).

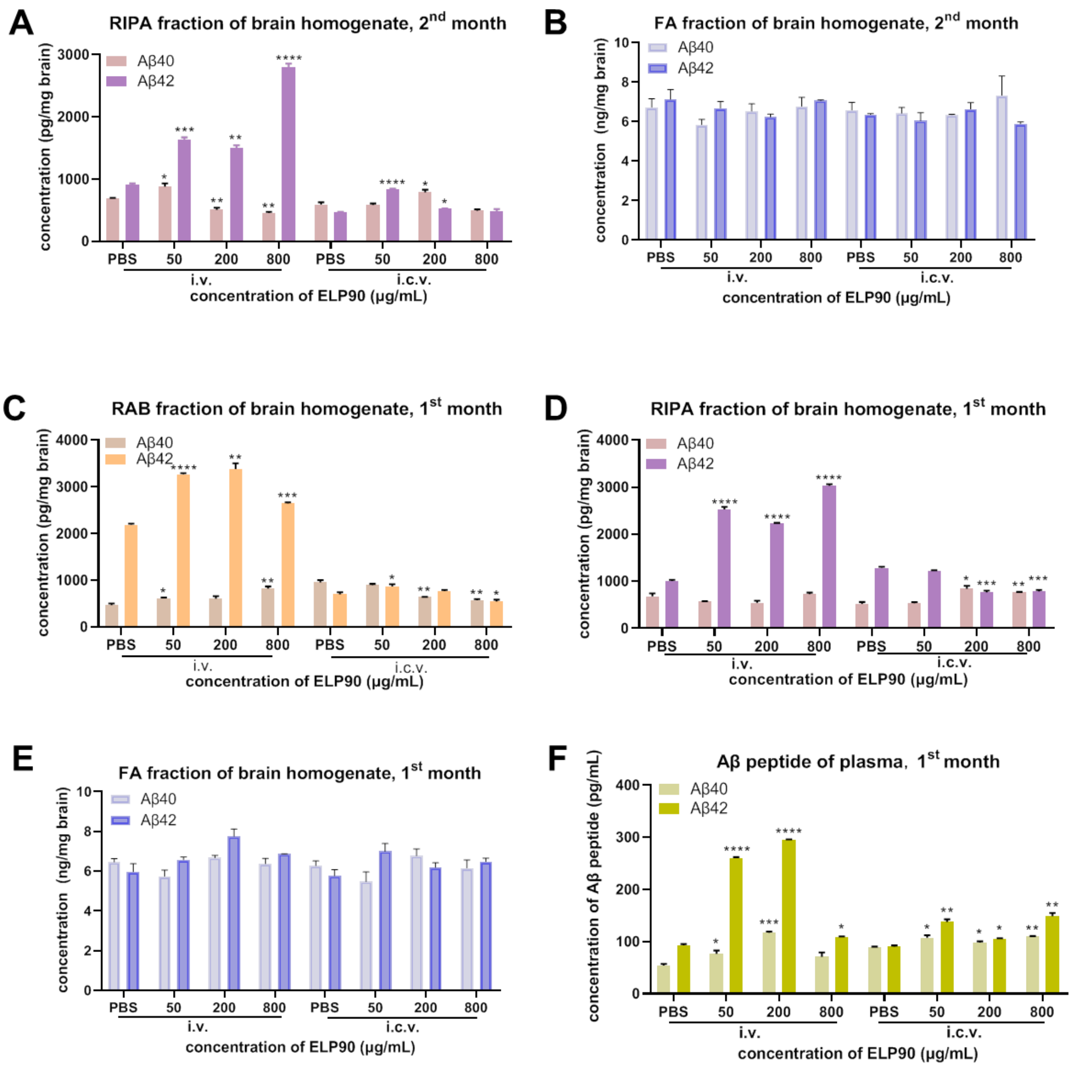

Figure S5. Concentration of Aβ40 and Aβ42 in brain homogenate and plasma. Proteins in brain samples were extracted with RAB buffer, RIPA buffer and FA buffer sequentially as described in methods ("Preparation of brain homogenate"), which resulted in a hydrosoluble protein fraction, detergent soluble protein fraction and insoluble fraction, respectively. The concentration of Aβ40 and Aβ42 in these three fraction of brain extract were detected with an ELISA kit, and calculated by the standard curve as described in methods. (A) Concentration of Aβ40 and Aβ42 in RIPA fraction in 2 month brains. In i.v. groups, compared with the PBS subgroup, Aβ42 showed a significant increase in all the ELP-treated groups, conversely, the Aβ40 showed a significant dose dependent decline in ELP-treated groups. The fold-change of Aβ42 was higher than that of Aβ40, suggesting an increase cleavage level of APP by β-secretase. Compared with PBS treated mice in i.c.v. groups, the levels of both the Aβ40 and Aβ42 in the RIPA fraction increased with low fold-changes. The concentration of Aβ42 and Aβ42 in 50μg/mL subgroups and the concentration of Aβ42 in 200μg/mL groups showed statistically significant differences compared with PBS subgroup. Data are presented as mean ± S.E.M. *$p<0.05$, **$p<0.01$, ***$p<0.001$, ****$p<0.0001$ by Students' t test. (B) Concentration of Aβ40 and Aβ42 in the FA fraction in the 2 month groups. Both the concentrations of Aβ40 and Aβ42 in all groups were in the ranges of 5.5 to 7.4. No significant

differences were seen among ELP- treated mice and PBS-treated mice in i.v. groups or i.c.v. groups. Data are presented as mean ± S.E.M. (C) Concentration of Aβ40 and Aβ42 of RAB fraction in 1 month mice. In the i.v. group, the concentration of Aβ40 showed little increase in three experimental groups, the 50μg/mL and the 800μg/mL showed statistically significant differences compared with the PBS group. The concentration of Aβ42 showed significant increase in all experimental groups compared with the PBS group, and the 200μg/mL subgroup had the highest concentration of Aβ42, which appeared to be a similar trend to that in 2 month (Fig.4D). In the i.c.v. group, both the concentration of Aβ40 and Aβ42 in the RAB fraction showed a decrease or limited increase in the three experimental groups with statistically significant differences. This may because of a delayed reaction to a low dose of ELP90 via i.c.v. administration in mice. Data are presented as mean ± S.E.M. *p<0.05, **p<0.01, ***p<0.001, ****p<0.0001 by Students' t test. (D) Concentration of Aβ40 and Aβ42 of the RIPA fraction at 1 month. The concentration changes of Aβ40 and Aβ42 in the i.v. group showed a similar tendency to that in the 2 month group. All three experimental groups demonstrated statistically significant increases as compared to the PBS subgroup. In the i.c.v. group, both Aβ40 and Aβ42 in the 200μg/mL and 800μg/mL subgroups showed a significant decrease compared with the PBS subgroup. Data are presented as mean ± S.E.M. *p<0.05, **p<0.01, ***p<0.001, ****p<0.0001 by Students' t test. (E) Concentration of Aβ40 and Aβ42 in the FA fraction in 1 month groups. Both of the concentrations of Aβ40 and Aβ42 in all groups were in the ranges of 5.4 to 7.4 ng/mg. Like the tendency shown in the 2 month group, there were no significant differences among ELP- treated mice and PBS-treated mice in the i.v. or i.c.v. groups. Data are presented as mean ± S.E.M. (F) concentration of Aβ40 and Aβ42 in plasma of the i.v. and i.c.v. groups at 1 month. In the i.v. group, the plasma Aβ42 level of all ELP-treated subgroups showed a statistically significant increase compared with the PBS group, and the 200μg/mL group showed the highest concentration of Aβ42. The plasma Aβ40 in all ELP-treated subgroups was also higher than that in the PBS group, and the 50μg/mL and 200μg/mL subgroups appeared to show statistically significant differences. In the i.c.v group, both Aβ40 and Aβ42 levels in the ELP-treated subgroups showed significant increase compared to the PBS subgroup. Data are presented as mean ± S.E.M. *p<0.05, **p<0.01, ***p<0.001, ****p<0.0001 by Students' t test.

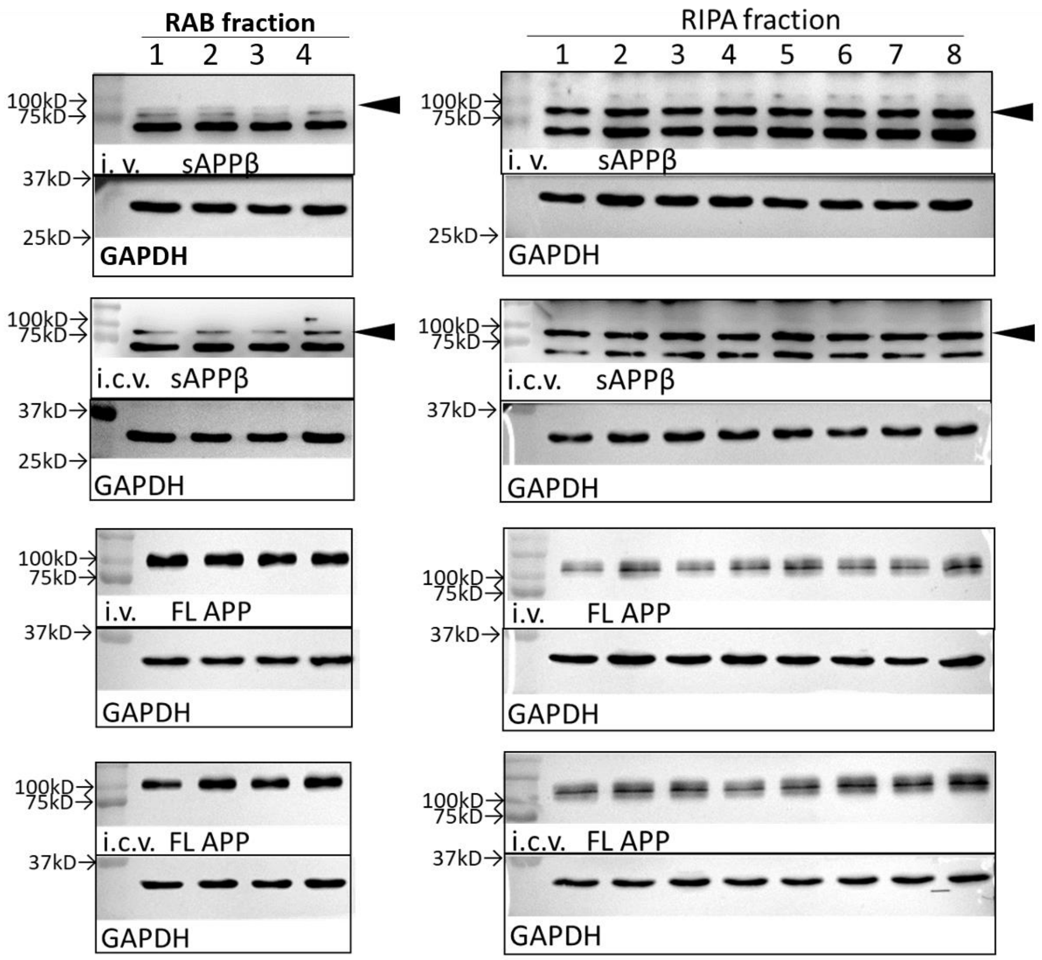

Relative quantity	RAB fraction				RIPA fraction							
lane	1	2	3	4	1	2	3	4	5	6	7	8
i.v sAPPβ	0. 10	0. 12	0. 08	0. 05	0. 67	0. 68	0. 67	0. 77	0. 92	1. 02	1. 03	0. 98
i.c.v. sAPPβ	0. 49	0. 64	0. 54	0. 64	0. 89	0. 81	1. 02	0. 87	1. 18	1. 28	1. 12	0. 94
i.v. FL APP	1. 36	1. 51	1. 34	1. 22	0. 60	0. 96	0. 64	0. 81	1. 25	1. 00	1. 16	1. 26
i.c.v. FL APP	0. 99	1. 33	1. 19	1. 20	1. 23	1. 61	1. 35	0. 82	1. 43	1. 83	1. 57	1. 87

Figure S6. Western blot of sAPPβ and full length (FL) APP from RIPA fractions of brain homogenate in i.v. and i.c.v groups. GAPDH act as internal control to measure the concentration of total protein in samples. The black arrow indicates the target bands. Lane 1 to lane 4 were brain samples in 1st month, lane 5 to lane 8 were brain samples in 2nd month. Lane 1/lane 5: PBS group, lane 2/lane 6: 50μg/mL group, lane 3/lane 7: 200μg/mL group, lane 4/lane 8: 800μg/mL group. In 1st month, sAPPβ showed limited increase in the ELP-treated subgroups administrated with i.v., but in the i.c.v group, the sAPPβ showed higher levels in the three ELP-treated subgroups. The FL APP did not appear to be different among all the subgroups. In addition, both the sAPPβ and FL APP showed no differences among all the groups in the RIPA fraction of brain homogenate from one month to two months. The quantification of staining lanes of sAPPβ and FL APP are shown in the table. In the bottom table, the numbers in red indicated the increase of sAPPβ in RIPA fractions of both i.v. and i.c.v. groups (lane 6) compared to control groups (lane 5).

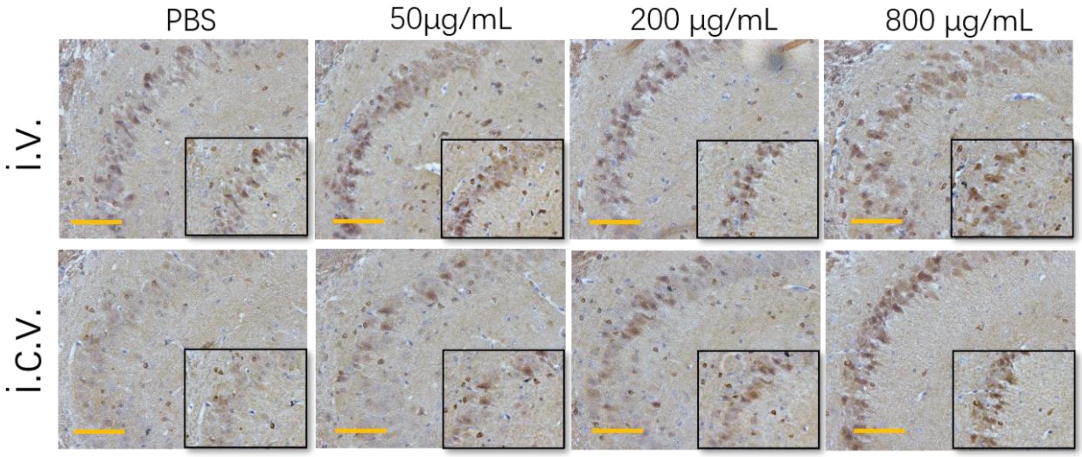

Figure S7. IHC of phospho-Tau (Ser396) in the CA3 region of hippocampus in mouse brains at 2 month after administration of different concentrations of ELP90. The figure shows the CA3 region and the thumbnails at bottom right show a double magnification of neurons in CA3. Phospho-Tau (Ser396) protein was recognized by PHF13 antibody and the brown pixels in slides represented the protein immunosignal.[4,5] There was an increased tendency of the phospho-Tau protein accumulating at the soma and dendrite of neurons as the concentration of ELP90 rose, suggesting that the hyperphosphorylation of Tau protein also participated in the pathological changes of ELP-treated mice. Scale bar: 0.2 mm.

Reference

[1] A. T. Welzel, J. E. Maggio, G. M. Shankar, D. E. Walker, B. L. Ostaszewski, S. Li, I. Klyubin, M. J. Rowan, P. Seubert, D. M. Walsh, D. J. Selkoe, *Biochemistry* **2014**, *53*, 3908-3921.

[2] S. S. Sisodia, P. H. St George-Hyslop, *Nat. Rev. Neurosci.* **2002**, *3*, 281-290.

[3] C. Zhang, P. J. Khandelwal, R. Chakraborty, T. L. Cuellar, S. Sarangi, S. A. Patel, C. P. Cosentino, M. O'Connor, J. C. Lee, R. E. Tanzi, A. J. Saunders, *Mol. Neurodegener.* **2007**, *2*, 15.

[4] E. Lauretti, L. Iuliano, D. Praticò, *Ann. Clin. Transl. Neurol.* **2017**, *4*, 564.

[5] J. Li, J. Chu, C. Barrero, S. Merali, D. Praticò, *Ann. Neurol.*, **2014**, *75*, 851.
